# Impact of Health Systems on the Implementation of Intermittent Preventive Treatment for Malaria in Pregnancy in Sub-Saharan Africa: A Narrative Synthesis

**DOI:** 10.3390/tropicalmed5030134

**Published:** 2020-08-22

**Authors:** Atinuke O. Olaleye, Oladapo Walker

**Affiliations:** 1 Department of Obstetrics and Gynecology, Babcock University, Ilishan 121103, Ogun State, Nigeria; 2 Department of Pharmacology and Therapeutics, Babcock University, Ilishan 121103, Ogun State, Nigeria; oladapo.walker@gmail.com

**Keywords:** malaria, pregnancy, intermittent preventive treatment, health systems, public health, sub-Saharan Africa

## Abstract

Malaria in pregnancy is a public health challenge with serious negative maternal and newborn consequences. Intermittent preventive treatment (IPTp) with sulphadoxine-pyrimethamine is recommended for the control of malaria during pregnancy within endemic areas, but coverage for the recommended ≥3 doses IPTp regimen has remained suboptimal. We searched PubMed, Cochrane library, and HINARI database from 1 January 2010 to 23 May 2020, for studies investigating the effect of the health system on IPTp implementation. Data extraction was independently performed by two investigators and evaluated for quality and content. Health system barriers and facilitators were explored using thematic analysis and narrative synthesis. Thirty-four out of 1032 screened articles were included. Key health system issues affecting the provision and uptake of IPTp were the ambiguity of policy and guidelines for IPTp administration, human resource shortages, drug stock-outs, conflicting policy implementation on free IPTp provision, hidden costs, unclear data recording and reporting guidelines, and poor quality of care. Factors affecting the supply and demand for IPTp services involve all pillars of the health system across different countries. The success of health programs such as IPTp will thus depend on how well the different pillars of the health system are articulated towards the success of each program.

## 1. Introduction

It is estimated that more than 25 million pregnancies will occur annually in malaria-endemic areas of the world, with more than 90% of these women being at risk for malaria infections each year [1]. The contribution of malaria to maternal mortality, preterm deaths, low birth weight babies, anemia in pregnancy, and neonatal mortality is well documented [1,2,3,4]. Underlying this heavy burden of malaria in pregnancy (MiP) is the generally high maternal and neonatal mortality in the African continent [5]. It has been further elucidated that there is a correlation between low birth weight and neonatal deaths, with malaria accounting for more than 10% of this burden [6]. MiP is thus a public health challenge, which has to be tackled robustly if we are to achieve the sustainable development goal (SDG) targets.

The obvious pharmacological approach to MiP control or prevention is the provision of prophylaxis. Prior, when *Plasmodium falciparum* was sensitive to chloroquine, the prophylactic regimen for pregnant women was a simple weekly administration of a stat dose of the full chloroquine treatment regimen [7]. Although there were variations to this approach in different malaria programs across the world, it was the gold standard of its era. Chloroquine resistance first appeared in Africa and Central America in the eighties, though it had been first described in Southeast Asia in the seventies [8,9,10,11]. Chloroquine was then replaced with the sulphadoxine-pyrimethamine (SP) compound, which became the new standard for intermittent preventive treatment in pregnancy (IPTp) [12,13,14,15].

Administration of IPTp with SP during routine antenatal care (ANC) and the use of insecticide treated nets (ITNs) are key strategies advocated for the control of malaria during pregnancy, in regions of stable transmission [3]. The efficacy of SP for the prevention of malaria in pregnancy was first demonstrated in Malawi [16,17,18]. Since then, the recommendation for IPTp has been modified from two treatment doses to three or more doses given at least one month apart, commencing in the second trimester of pregnancy [12,18,19]. The WHO currently recommends IPTp-SP in all areas with moderate to high malaria transmission in Africa. There is insufficient evidence to support a general recommendation for its use outside this region [3]. Breakthrough malaria infections are treated with the standard drug regimen as recommended by each country’s antimalarial treatment policy.

Although this approach to the prevention of malaria in pregnant women appears to be simple, optimal coverage for the ≥3 doses IPTp regimen has been a great challenge. Adherence to the IPTp regimen appears to decrease despite an increase in the number of ANC visits [20,21,22,23,24,25]. In 2018, the coverage rate for three doses of IPTp globally was 31% [26].

To achieve the malaria elimination agenda, it is important to ensure the successful implementation and continuity of proven multi-sectoral strategies at community, regional, and global levels. This, however, requires a functional health system to anticipate, detect and/or mitigate challenges expected with such approaches, as they are often affected by multi-faceted social, cultural, economic, and institutional factors. The WHO framework for the health system is built on six pillars: service delivery; health workforce; health information; medical products, vaccines and technologies; healthcare financing; leadership and governance [27]. These are critical vehicles for facilitating the implementation of interventions to improve maternal and neonatal health. It becomes important, therefore, to evaluate the impact of the health system on the implementation effectiveness of the IPTp strategy, as we work towards the goal of malaria elimination.

To the best of our knowledge, there are scant data on the holistic evaluation of the influence of the health system on IPTp implementation. This was the major reason for the review. This study would therefore add value to the existing body of knowledge and provide recommendations towards achieving malaria elimination.

## 2. Methods

The following search engines were used to undertake an electronic systematic literature search from 1 January 2010 till 23 May 2020: PubMed, Cochrane library, and HINARI. Although Google Scholar was used in extracting some of our references, these largely overlapped with our primary source. Studies conducted between 2010 and 2020 were selected because the WHO monograph on the health system was written in 2007, and it takes about two years for countries to complete the process of revising their IPTp policies [28] before cascading to the sub-national levels. A combination of keywords and medical subject headings (MeSH terms) were used to identify studies assessing the effect of the health system on IPTp implementation. Attempts were made to identify all relevant studies in the English language without prejudice to publication status. The reference lists of retrieved studies were also reviewed for additional relevant studies. The Preferred Reporting Items for Systematic reviews and Meta-Analyses (PRISMA) guidelines and flow diagram [29] were used to report the search and selection of studies. The keywords, inclusion, and exclusion criteria are as stated below:

1. Keywords: health systems; malaria in pregnancy; intermittent preventive treatment of malaria.
2. Inclusion criteria:
  - Studies that describe the health system and investigate malaria in pregnancy
  - Studies that investigate the effect of the six pillars of the health system and IPTp
  - Studies investigating the outcomes of malaria in pregnancy within the health system
  - Studies published in English language between 2010 and 2020
3. Exclusion criteria:
  - Studies outside the date range
  - Studies on malaria that do not address malaria in pregnancy

Two review authors (O.W. and A.O.) independently screened the titles and abstracts of literature search results for potentially relevant studies and extracted the full articles of these studies. The eligibility criteria were independently applied to the articles and the publications were scrutinized to ensure each study was included in the review only once. Where there were doubts about eligibility, a discussion was held among the principal authors to resolve the differences. A thematic analysis was conducted, and the findings were presented using the narrative synthesis approach, which uses a textual approach in summarizing review findings [30]. One of the authors (O.W.) coded the tabulated findings on the review areas using common codes, concepts, and categories, while A.O. categorized and synthesized emergent codes into the final themes. Both authors reviewed all synthesized findings to identify areas of convergence and divergence and resolved any disagreements, ensuring findings reflected the areas under review.

## 3. Results and Discussion

**Figure 1:**
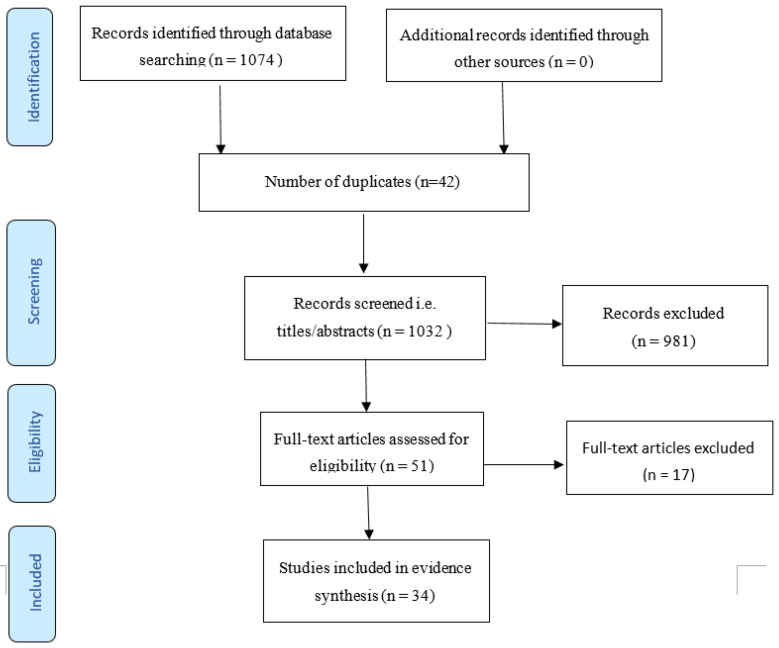
PRISMA flow diagram of identified studies.

The search output identified 1074 references in total, of which 42 were duplicate reports. Out of the 1032 remaining titles following removal of the duplicate reports, 981 titles and abstracts were screened out. The full-text articles of 51 studies were retrieved for eligibility screening, after which 34 studies met the inclusion criteria for this review. A flow diagram of the study selection process is shown in Figure 1. The characteristics of the included studies are highlighted in Table 1.

The studies included in this review comprised observational (22) and interventional (2) quantitative studies conducted among pregnant women and healthcare providers across sub-Saharan Africa. Qualitative (8) and mixed-method (2) studies exploring provider and client perspectives on the barriers to IPTp uptake were also reviewed. The studies were generally of good quality, based on the National Institutes of Health (NIH) quality assessment tool for observational cohort and cross-sectional studies [58]. Parameters appraised include the clarity of the research question, appropriateness of the study population and sample size, outcome assessment and data analysis. Appraisal of the qualitative studies was carried out using the quality assessment tool described by Lorenc et al. [59], which consists of nine questions. The results were adjudged good, fair or poor. Table 2 highlights the location of the various publications within the regions of sub-Saharan Africa.

**Figure 2:**
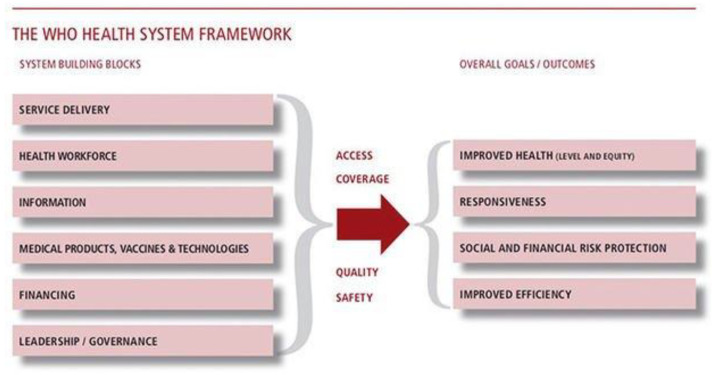
With permission from Everybody’s business: strengthening health systems to improve health outcomes. WHO’s Framework for Action, 2007 [27].

The WHO in a seminal publication defined Health System as “Everybody’s Business”. It consists of all those elements that work together as a whole to improve health outcomes. This framework is built on six pillars: service delivery; health workforce; health information; medical products, vaccines and technologies; health financing; leadership and governance [27]. The interaction and interdependence of these building blocks are necessary to ensure improved health access, coverage, quality and safety (Figure 2).

A general observation in this review is that not all the studies from the various countries emphasized the different pillars of the health system in the context of IPTp. While one review might focus on the health workforce, others might seem to examine the information system. Thus, at first glance, it might appear that the review examined a particular pillar and not the whole system. We believe that the data provided was largely due to what was obtained on the ground during the investigations, and that countries are working hard to improve their systems. However, the success of health programs will depend on how well the different building blocks of the health system are articulated towards the success of each program.

It is important to note that though it is located within the malaria program, IPTp implementation is dependent on existing maternal health services. Hence, the findings in this review reflect health system issues in the context of the maternal health and malaria programs. A summary of the health system challenges for IPTp implementation from the studies reviewed are shown in Table 3.

**Service Delivery**: This building block focuses on the organization and management of health services in an equitable manner to ensure access, safety, quality and adequate coverage [27]. This review demonstrated some serious provider challenges with the implementation of IPTp, as there were issues such as human resource gaps, work overload, poor working conditions, and client challenges that stressed the healthcare workers [43,46,52,53,56,57]. Some authors attributed much of the above to health system defects [42]. Training of ANC providers on IPTp delivery appears to have the most significant potential impact for increasing IPTp coverage, as some authors suggest that health provider practices rather than women’s ANC attendance are primarily responsible for the ineffectiveness of the IPTp strategy [36,51].

Note is taken of the long delay in the implementation of IPTp when changes were made in the doses to be given in Malawi [29]. This delay negatively affects health service delivery generally, as the health workers need guidelines from policy documents to deliver the services that clients require. A major contributor to the ineffectiveness of the IPTp strategy is missed opportunities at health facilities during women’s ANC visits [51,60]. Location and accessibility of healthcare facilities were related to IPTp uptake, with longer distances associated with reduced uptake of IPTp [33,35,55,56]. Perceived poor quality of care is a barrier to ANC utilization, thereby reducing opportunities for IPTp administration [35]. Ensuring sustained demand for IPTp is important in achieving the goal of reduction in malaria in pregnancy, and this will require effective service delivery mechanisms that are responsive to client needs and expectations.

**Health Workforce**: This review confirmed existing knowledge about the weak health workforce in much of the African health systems [34]. Several studies reported poor health worker knowledge of IPTp guidelines/protocols, uncertainty about the safety and efficacy of SP, as well as inadequate and unsystematic supervision [20,23,32,33,38,47,57]. General concerns affecting job satisfaction include poor working environment and limited career development opportunities [43,57]. Understaffing is a key issue, and human resource shortages are aggravated by a mismatch of expertise among the available personnel.

The challenge of weak policy for the health workforce trickles down to the programs. Thus, poor capacity building on the job, improper deployment of personnel and poor quality control measures all conspire to produce poor output from healthcare personnel, even where such persons mean well [49]. Health worker encouragement is one of the factors positively influencing pregnant women’s demand for ANC and IPTp services [50]. Health workers also play a role in addressing several factors that negatively influence client demand, such as lack of knowledge about MiP, late initiation of ANC, reluctance of pregnant women to take medication and fear of the effects of drugs on the unborn child [21,37,43,45,50,52,54].

The factors that beleaguer the health workforce in the African region are amply demonstrated in some of the studies that showed a number of the weaknesses enumerated above [33,57]. There is therefore rationale for the various health systems to conduct a scoping review of their health workforce regularly, in order to produce local solutions for local problems. In addition, care must be taken to examine the demand and production sides of the health workforce to introduce a good balance on the production side. This calls for in-depth policy analysis and change, if the health system in the African region is to make progress towards local and international goals [39].

**Health Information**: The health information system generally comprises three domains: information on health determinants; health system performance; health status [27]. The data obtained in this review showed several weaknesses in the information systems across the three domains as they are being operated currently [33]. Although data are often generated using various standardized instruments and tools within countries at both population and facility level, there is a capacity gap in the synthesis, application and dissemination of the information obtained. Most data obtained from the facilities are documented in handwritten logbooks, with a tendency for errors during transcription into electronic platforms. Inaccuracies and other issues in data recording and reporting often result in an insufficient response of policymakers to emerging trends and challenges [32,33]. In several malaria indicator surveys, it is noted that IPTp was one of the important indicators [31,40]. This is not surprising as malaria in pregnancy is an important focus of malaria elimination. However, utilization of data points in facility ANC records to address issues such as missed opportunities for IPTp among ANC attendees at the district level are often lacking, as a comprehensive preliminary analysis of the data obtained by the district authorities for local use, is not frequently done. Usually, data collected are transmitted centrally, with little or no feedback to the facilities that generate the data. In addition, findings from data obtained by partners during various program assessments are often sent to their principals without inputs into practices at the district level. Where such data get transmitted to the government, there is usually no ownership of the data centrally. Opportunities for health system strengthening are therefore missed.

What is currently required is not only the acquisition of data, but intelligent use of the information gathered, for progress towards malaria elimination. Continuous capacity building of all cadres of the health workforce to keep abreast of developments locally and globally towards this goal is thus essential. There should be discussions between the field workers, program implementers and policymakers to ensure optimum use of the data. This triangulation of discussions will help to ensure appropriate synthesis and application of data for policy decisions.

**Medical Products**: A functional health system ensures access to and use of essential medical products of assured quality, safety, efficacy and cost-effectiveness in a rational and equitable manner. It was observed that there was generally a lack of standardization concerning the administration of IPTp [33], with large variations in IPTp administration, especially with directly observed therapy (DOT). When hospitals were compared to health centers, the health centers performed better. The level of implementation of DOT ranged from 0 to 53%, with health centers having the higher level of implementation [42]. Compliance with IPTp protocol appeared to be highest at the community level [38,60]. One obvious theme that emerged in the studies was that some health workers were not very proficient with the administration of the medications. This could be due to faulty training, and the inability of the supervisors to perform proper quality assurance and monitoring. Indeed, in one of the studies, there was a complaint about overbearing supervisors and system challenges with administration of IPTp [57].

There were also seemingly minor but important reasons for the improper implementation of IPTp, such as distance from the health center, absence of potable water at the health center level for drug administration by DOT, inadequate education of the client about IPTp and drug side effects [25,41,53]. Within the health system, this type of situation leads to a domino effect, with several lapses piling up and resulting in system failure for a particular program.

Drug stock outs associated with the slow allocation of quarterly funds to the district and beyond was a constant observation in the studies reviewed [20,25,33,38,43,56,57]. Drug stock outs have been frequently reported during monitoring and evaluation of programs. As malaria is an important underlying cause for under-five mortality in Africa, it is peculiar that health facilities would run out of antimalarial drugs [48]. It is therefore clear that the supply chain has to be more robust, to take care of priority programs that affect millions of people in endemic areas.

**Health Financing (Costs)**: A good health financing system requires a sustainable financing mechanism that protects people from catastrophic health expenditures [27]. Universal health coverage is advocated to promote equity and access to health services for all. However, its implementation is a challenge for many countries, particularly for those in sub-Saharan Africa where out-of-pocket payments are very high [44]. In countries where funding exists for ANC (and IPTp) services, implementation challenges still occur. In Tanzania, bureaucratic bottlenecks in the approval and disbursement of funds from the national level often impair the implementation of ANC (and IPTp) activities at district health facilities [43].

It was evident from the review that the poorer women in most countries were unable to comply with the required number of ANC visits and IPTp doses, as IPTp uptake was related to wealth index—the higher the client was in the socioeconomic group, the more likely she was to comply with IPTp uptake [40,55,61,64,65]. Health insurance was also shown to be an important factor in complying with IPTp [65]. When the issue of cost was eliminated, studies reported generally good IPTp uptake, as the drugs were received free and sometimes delivered to their homes [66,67]. This was possible because the program was performed under quasi-experimental conditions, and the costs of the drugs were embedded into the project costs. However, hidden costs such as transportation fees, time off work and purchase of drugs from the private market due to stock-outs at health facilities still exist and are important deterrents to the uptake of IPTp [68] and other maternal health services.

Where inconsistencies in policy implementation regarding IPTp provision occur, uptake remains poor. For instance, in Mali the government policy states that IPTp is free, but health facilities still charge for it as part of user fees to generate operational revenue [63].

The financial inputs of governments in Africa into the health sector are well below what is expected by international standards [49,69], with inadequate social and financial protection mechanisms resulting in the majority of the people paying out of pocket for healthcare [44]. Over 400 million people (>40% of the population) in sub-Saharan Africa live below two dollars a day [70]. Given the foregoing, costs of services are issues of prime importance if we intend to bring services to these people. The primary healthcare (PHC) concept developed by the WHO to ensure equity and accessibility to care [71,72], is facing issues of sustainability in many African countries. To mitigate this, supportive programs such as community-based health insurance may be considered or strengthened where it already exists, to improve access of poor rural households to quality health services and reduce catastrophic health expenditure [73,74].

**Leadership and Governance**: It is no surprise that there are challenges with the implementation of IPTp in the African region, as highlighted in previous reviews [62,75]. A major leadership challenge is in policymaking and implementation. Ambiguity of policy and guidelines for IPTp administration, such as number and timing of doses, gestational age limits and DOT implementation predispose to ineffective service delivery with patchy IPTp uptake [32,38,53]. Programs whose ownership cuts across several agencies, such as IPTp that involves maternal health and malaria programs, are at risk of dichotomy in management. Studies show differences in the coordination between government health authorities and program implementation partners, even within the same country [32]. This could create a gap in the capacity building, monitoring and evaluation of such programs, as the non-governmental stakeholders usually carry out these roles.

A key issue also identified is the absence of strong links between the malaria program and other disease-specific programs involved with maternal ANC services, such as HIV and immunization [32]. Inadequate stakeholder coordination and linkage of such related programs will lead to fragmented service delivery, with resultant patchy implementation [67]. It is obvious that at the country level, policymakers, implementers, and the communities must all be on the same page if we hope to make our policies work, at the right time. Policymakers must bridge the gap in translation of policy to implementation as was highlighted by a Ugandan study, where IPTp guidelines did not reflect the most recent WHO policy recommendation [32], and a Tanzanian study showed that two different agencies were busy implementing the IPTp policy as they saw fit [53,57]. Situations like this demonstrate quality assurance and policy enforcement weaknesses, which may lead to poor program performance. It is suggested that when the policy holders (in this case the malaria program) are in the process of drawing up their implementation plans, the major stakeholders must be on the same page with the technical arm of the program.

There is a need for countries to devise and embrace evidence-based innovative approaches to improving IPTp uptake, following consideration of their operational feasibility, scalability, and applicability to their settings. This would include approaches that address bottlenecks identified within their own health system to reduce barriers to access, quality, safety, and coverage. Such approaches may involve leveraging on digital platforms such as mobile phone messaging to provide appropriate information on antenatal care (including IPTp) and reminders for ANC visits [76], home delivery of IPTp drugs to pregnant women [66] and sustainable means of health financing.

## 4. Conclusions

It is evident from this review that there are serious health system lapses that contribute to the low uptake of IPTp. These include poor training of workers, inadequate provision of simple tools for work, poor record-keeping in the facility, poor data entry into the health information management system, and drug stock outs. It appears that all pillars of the health system have been affected in some manner in different countries. The systemic issues identified are reflective of the IPTp services, but more importantly, they highlight the situation of existing maternal health and malaria programs within the countries. It is vital that the gaps elucidated within the health system framework be addressed in these programs, as improvement in the effectiveness of both the maternal health and malaria programs are likely to positively influence IPTp uptake.

Capacity for the collection, synthesis, interpretation, and dissemination of health-related data should be strengthened at national and local levels. Evidence-based, context-sensitive innovations for improving IPTp uptake should be evaluated and adapted, if suitable to the country setting. Accurate and comprehensive data will help countries to identify and prioritize the health system pillars that require interventions for improved effectiveness of the health system in general. It will also help policymakers, program managers and health administrators to make appropriate decisions on practical issues involving maternal health service delivery, health workforce capacity and distribution, drug availability and quality, health financing mechanisms, and health policy in a timely and accurate manner.

## Figures and Tables

**Table 1:** Characteristics of included studies.

Author	Year	Country	Setting
Ameh [31]	2016	Nigeria	Cross-sectional study of 400 ANC attendees to identify barriers to and determinants of IPTp uptake
Arnaldo [32]	2019	Mozambique	Qualitative study on access to and use of preventive intermittent treatment for Malaria during pregnancy
Arnaldo [33]	2018	Mozambique	Community and facility-based surveys of 1141 mothers to assess IPTp-SP coverage and factors associated with low uptake
Awantang [34]	2018	Madagascar	Cross-sectional household survey of mothers with children under the age of 2 years on factors associated with IPTp uptake
Azizi [35]	2020	Malawi	Analysis of 2015–16 DHS dataset to assess uptake of IPTp in Malawi after adoption of updated WHO IPTp-SP policy
Buh [36]	2019	Sierra Leone	Secondary data analysis of the Multiple Indicator Cluster Survey (MICS 5) among reproductive age women
Dellicour [37]	2016	Kenya	Cross-sectional facility survey on effectiveness of the delivery of interventions to prevent malaria in pregnancy
Diala [19]	2013	Nigeria	Qualitative study on perceptions of IPTp and barriers to adherence
Henriksson [38]	2017	Uganda	Secondary data analysis to identify bottlenecks in service delivery within the district health system
Henry [28]	2018	17 countries in sub-Saharan Africa	Country analysis of implementation of updated WHO policy
Hill [39]	2013	Kenya	Secondary analysis of household survey data to evaluate the effectiveness of antenatal clinics to deliver IPTp and ITNs
Hill [40]	2014	Mali	Secondary analysis of household survey data to evaluate the effectiveness of antenatal clinics to deliver 2 doses of IPTp and ITNs
Hill [41]	2016	Kenya	Qualitative data on user and provider acceptability within RCT comparing ISTp-DP and IPTp-DP with IPTp-SP
Iliyasu [20]	2012	Nigeria	Cross-sectional study of 239 ANC attendees on IPT adherence
Kibusi [30]	2015	Tanzania	Analysis of 2011–2012 HIV and Malaria Indicators Survey of 1616 women to identify factors responsible for IPTp uptake
Kibusi [42]	2018	Tanzania	Analysis of 2011–2012 HIV and Malaria Indicators Survey to examine the role of health insurance coverage in utilization of maternal health services
Klein [43]	2016	Mali	Qualitative study of pregnant women, husbands, mothers-in-law and health workers in two rural regions
Konje [44]	2018	Tanzania	Mixed method population-based study on ANC availability, utilization, challenges and barriers among pregnant women and community health workers in rural areas
Maheu-Giroux [45]	2014	Kenya, Namibia, Rwanda, Tanzania, Uganda	Secondary data analysis of service provision assessment surveys over a five-year period to explore factors affecting provider’s delivery of IPTp during ANC consultations
Mbengue [46]	2017	Senegal	Secondary analysis of 4616 women from 2013–2014 Demographic and Health Survey to assess factors associated with optimal IPTp and ITN uptake
Mubyazi [47]	2014	Tanzania	Mixed method study among ANC providers in two rural districts on psychosocial, behavioural and health system barriers to IPTp delivery and uptake
Mubyazi [48]	2013	Tanzania	Qualitative study among health managers in public and private clinics on IPTp feasibility, acceptability and challenges
Mubyazi [49]	2012	Tanzania	Qualitative study on the drivers of motivation and performance among health workers providing IPTp in public and private facilities
Okello [50]	2018	Kenya	Qualitative study among health workers and sub-country managers
Okethwangu [51]	2019	Uganda	Secondary data analysis from Uganda Demographic and Health Survey on factors associated with uptake of optimal IPTp doses
Onoka [22]	2012	Nigeria	Cross-sectional study among healthcare providers
Onyebuchi [23]	2014	Nigeria	Prospective descriptive study of 516 pregnant women from their ANC booking till delivery
Oppong [52]	2019	Ghana	5-year retrospective data analysis of a district within a Health and Demographic Surveillance System area
Orobaton [53]	2016	Nigeria	Community intervention study on IPTp delivery strategies to assess scalability, costs and program impact
Rassi [54]	2016	Uganda	Qualitative study of district health officials, health workers, pregnant women and opinion leaders (supply side)
Rassi [55]	2016	Uganda	Qualitative study of district health officials, health workers, pregnant women and opinion leaders (demand side)
Toure [24]	2014	Cote D’Ivoire	Cross-sectional survey of 1317 delivered women
Yaya [56]	2018	Burkina Faso, Ghana, Mali, Malawi, Kenya, Nigeria, Sierra Leone, and Uganda	Cross-sectional data on 18,603 women from Malaria Indicator Surveys
Webster [57]	2013	Mali	Cross-sectional study of 780 women to evaluate health system effectiveness of ANC for the delivery of a dose of IPTp and an ITN

**Table 2:** Location of included studies.

Sub-Saharan Africa Region	Number of Publications
East Africa	19
West Africa	12
Southern Africa	0
Trans-regional publications	3
Total	34

**Table 3:** Studies on health system barriers to intermittent preventive treatment (IPTp) implementation.

Health System Pillar	Country	Barrier to IPTp Implementation
Service delivery	Kenya, Nigeria, Tanzania, Mali, Senegal	Ambiguity of IPTp policy and administration [33,53], poor supervision [47], poor quality of service [35,56], long distance [35], poor health worker adherence to IPTp guidelines including gestational age and DOT [38,42,60], poor provider knowledge of IPTp protocol [20,23,38], long waiting time [35], water shortages [43], SP safety concerns [38,53], informal health center regulations [35], complex guidelines [38], late ANC registration [53,60,61,62], insufficient time for ANC counseling [46]
Health workforce	Mozambique, Uganda, Kenya, Tanzania	Human resource shortages [33,35,52,56] understaffing [43,57], poor supervision [57], health worker job dissatisfaction [57]
Health information	Kenya	Ambiguity of IPTp data recording and reporting guidelines [33], redundant tools [33]
Medical products, vaccines and technologies	Kenya, Nigeria, Tanzania, Namibia, Rwanda, Uganda	Drug stock-out [20,33,35,36,47], absent or inadequate supply of free SP for IPTp to private facilities [43]
Healthcare financing	Mali, Tanzania, Ghana, Senegal	Out-of-pocket payment for healthcare [35,38], hidden costs [35], poverty [35,55,62], ambiguity on free and fee-based services at health facility [53,63], delayed government funding for health centers [43]
Leadership and governance	Mali, Mozambique	Inconsistent policy implementation [63], conflicting guidelines [53], poor community awareness of IPTp [52]
